# Intracapsular Tonsillectomy Using Plasma Ablation Versus Total Tonsillectomy: A Systematic Literature Review and Meta‐Analysis

**DOI:** 10.1002/oto2.22

**Published:** 2023-02-17

**Authors:** Matthew J. Sedgwick, Christopher Saunders, Neil Bateman

**Affiliations:** ^1^ Global Clinical and Medical Affairs Smith+Nephew Hull UK; ^2^ Paediatric Otolaryngology Department, Manchester Academic Health Science Centre, Royal Manchester Children's Hospital Manchester University Hospitals NHS Foundation Trust Manchester UK

**Keywords:** obstructive sleep apnea, pain, posttonsillectomy hemorrhage, tonsillectomy, tonsillitis, tonsillotomy

## Abstract

**Objective:**

To determine whether intracapsular tonsillectomy, using plasma ablation, results in differences in postoperative patient outcomes to total tonsillectomy.

**Data Sources:**

A systematic review of two databases (Embase and PubMed) was conducted in March 2022 to identify published English‐language randomized controlled trials and observational studies which provided a comparison between intracapsular tonsillectomy, using plasma ablation, and total tonsillectomy.

**Review Methods:**

Qualitative synthesis and meta‐analysis were used to compare outcomes between techniques.

**Results:**

Seventeen studies were identified for inclusion. Across these, 1996 and 4565 patients underwent intracapsular and total tonsillectomy, respectively. Studies included 8 randomized controlled trials, 1 prospective cohort study, and 8 retrospective cohort studies. Time to pain free, time on analgesia, time to normal diet, and time to normal activity were significantly shorter with intracapsular tonsillectomy by on average 4.2 (95% confidence interval [CI] 1.5‐5.9; *p* < .0001), 4.1 (95% CI 2.7‐5.4; *p* < .0001), 3.5 (95% CI 1.7‐5.4; *p* = .0002) and 2.8 (95% CI 1.6‐4; *p* < .0001) days, respectively. Risk of posttonsillectomy hemorrhage was significantly lower following intracapsular tonsillectomy (relative risk [RR] 0.36; 95% CI 0.16‐0.81; *p* = .0131); risk of posttonsillectomy hemorrhage requiring surgical management was lower but failed to reach significance (RR 0.52; 95% CI 0.19‐1.39; *p* = .19).

**Conclusion:**

Intracapsular tonsillectomy using plasma ablation has similar efficacy in managing indications for tonsil surgery to total tonsillectomy while significantly reducing the postoperative morbidity and likelihood of posttonsillectomy hemorrhage experienced by patients, allowing them to return to their normal life faster.

Tonsillectomy is commonly performed in both adult and pediatric populations primarily for obstructive sleep apnea and recurrent tonsillitis.^1^, ^2^ Traditionally, the default technique for tonsillectomy has involved the dissection of the pharyngeal tonsils from the muscles of the “tonsil bed” or the pharyngeal constrictor. This specific tonsillectomy procedure is often referred to as an “extracapsular” or “total” tonsillectomy. Total tonsillectomy is associated with several complications, including posttonsillectomy hemorrhage (PTH) (both primary and secondary) and postoperative pain.^3^, ^4^, ^5^, ^6^ These complications may result in a reduction in oral intake, dehydration, and readmission to hospital while also disrupting the child's and their parent's/guardian's/caregiver's normal lifestyle. Strategies that reduce these complications may therefore be of value by reducing the postoperative burden on the healthcare system and the child and their family.

The innervation of tonsil tissue with pain fibers is very much less than that of the pharyngeal musculature, and the diameter of blood vessels within the tonsil is less than those within the muscle. It, therefore, seems reasonable to contend that an avoidance of muscle exposure should reduce both postoperative pain, expediting the return to normal lifestyles, and the risk of PTH associated with total tonsillectomy. Consequently, in recent years attention has focused on the use of “partial” tonsillectomy procedures which avoid pharyngeal muscle exposure. Partial tonsillectomy techniques include a range of different surgical techniques that encompass a spectrum from tonsillotomy, which preserves some tonsil tissue (typically removing 50% of tonsillar tissue), to intracapsular tonsillectomy, which preserves just the fibrous capsule (typically removing >90% of tonsillar tissue).^7^ Large case series have shown efficacy of such procedures in both obstructive and infective indications while also demonstrating low rates of return to the operating room due to PTH and low postoperative morbidity associated with pain.^8^, ^9^, ^10^

Previous systematic literature reviews have explored the evidence comparing total tonsillectomy to all partial tonsillectomy techniques combined, and not intracapsular tonsillectomy specifically.^11^, ^12^, ^13^, ^14^, ^15^ However, it has been proposed that intracapsular tonsillectomy may offer additional patient benefits over tonsillotomy.^8^, ^10^ In addition, due to the growing popularity and recognition of a plasma ablation technology in tonsil procedures,^2^ the aim of this study was to perform a systematic literature review to determine whether intracapsular tonsillectomy performed using plasma ablation specifically resulted in differences in postoperative patient outcomes compared to total tonsillectomy procedures utilizing any device. The outcomes relating to pain, return to normal, and complications were assessed.

## Methods

This review was performed and written in accordance with Preferred Reporting Items for Systematic Reviews and Meta‐Analyses guidelines.^16^

### Search Strategy

A systematic literature review was performed using PubMed and Embase databases in March 2022 to identify articles of potential relevance evaluating intracapsular tonsillectomy using plasma ablation. The search strategy incorporated the search string of “(Coblation OR Bipolar OR Radiofrequency) AND (Tonsil*)” without any limitations or restrictions. No limits on publication year were implemented. Reference lists of included studies were also reviewed to identify articles for inclusion.

### Study Screening

Articles identified during the execution of the search strategy were screened for suitability by 2 independent reviewers, first based on the title and abstract and second based on the full text. Inclusion criteria consisted of a full‐text publication in the English language of primary clinical evidence in the form of a comparative clinical trial. The comparison was required to include 1 study group undergoing intracapsular tonsillectomy using plasma ablation (CIT; Coblation; Smith+Nephew) and the remaining study group(s) undergoing total tonsillectomy for obstructive and/or infective indications, with each study group containing over 5 patients, and reporting on the identified primary or secondary outcomes.

Intracapsular tonsillectomy was defined according to Windfuhr and Werner^7^ such that there was clear intention by the surgeon to remove the majority of the tonsil tissue. Where insufficient information on surgical technique was provided to classify the procedure as intracapsular, and in cases where relevant technique data were pooled with other tonsillectomy techniques, the article was excluded. The tonsillectomy was expected to be the primary procedure; however, concurrent adenoidectomy was not a reason for exclusion. Further to this, radiofrequency somnoplasty of the tonsils was not considered as a relevant control procedure^7^ and these studies were excluded.

### Data Extraction

Data associated with the study, patient and surgical procedure characteristics, along with outcome data were extracted into a predefined data extraction table by 1 reviewer and checked for accuracy by a second reviewer.

Study characteristics included author name, year of publication, study design, and sample size. Patient characteristics included age at time of surgery and indication for surgery. Surgical characteristics included type of device used in the total tonsillectomy group.

Postoperative outcomes of interest were: efficacy; absolute pain; time to pain free; time on analgesia; time to return to normal diet; time to return to normal activity; PTH rates (including all PTH, primary PTH, and secondary PTH and those specifically requiring a return to theater); and other complications. The incidence of events for binomial outcome variables and measure of central tendency and spread for continuous outcome variables were extracted. In cases where data was only available in the form of figures, this was extracted through electronic digitization (https://automeris.io/WebPlotDigitizer), a method described and validated by others.^17^, ^18^

### Quality Assessments

Each included study's risk of bias was assessed. For randomized controlled trials (RCT), the Centre for Reviews and Dissemination's guidance for the assessment of risk of bias in RCTs was followed.^19^ For observational studies, the Critical Appraisal Skills Program guidelines was followed.^20^

### Data Synthesis and Meta‐Analysis

Meta‐analysis was performed in R (version 4.0.2; R foundation for Statistical Computing) using the “meta” package, for comparison of outcomes between the experimental procedure (CIT) and the control procedure. For binomial outcomes, a relative risk (RR) with a 95% confidence interval (CI) was reported as the summary statistic and for continuous variables a mean difference (MD) (if reported on the same scale) or standardized mean difference (SMD) using Hedges *g* correction (if reported on difference scales) was reported with a 95% CI as the summary statistic. Absolute pain scores were analysed at the 3 most frequent time domains of 1 day, 1 week (5‐8 days), and 2 weeks. Heterogeneity of included studies was assessed using the *I* ^2^ statistic, the fixed effect model was utilized when *I* ^2^ < 50% and the random effects model when *I* ^2^ ≥ 50%. Forest plots support each meta‐analysis to visually display individual studies and heterogeneity within each synthesis. Sensitivity analysis was carried out by comparison of fixed effect and random effects outcomes, through leave‐one‐out analysis and substitution of equivalent data provided by patient or parent/guardian/caregiver. Additional qualitative synthesis was provided for studies which reported on a related outcome or when insufficient data were available preventing the use of meta‐analysis.

Where data were missing within papers (mean or standard deviation), techniques used by others to obtain missing data were followed. When a median was presented with no spread of data, the median was considered equivalent to the mean when sample size was >25.^21^ When a median was provided along with a range or interquartile range, the mean and standard deviation was determined using the estimates presented by Wan et al.^22^ Finally, when no measure of spread was provided a standard deviation was estimated based on the pooled standard deviation of similar studies.^23^ Where frequency for ordinal scales of pain were available (eg, low, moderate, and severe pain), these were transformed to quantitative scores with a score of 1 being assigned to the lowest level of pain and the resulting mean and standard deviation determined.

Analyses were considered statistically significant at the level of *p* < .05 and continuous data are presented as mean (standard deviation) and dichotomous data as events (% of study group) throughout unless otherwise stated.

## Results

**Figure 1:**
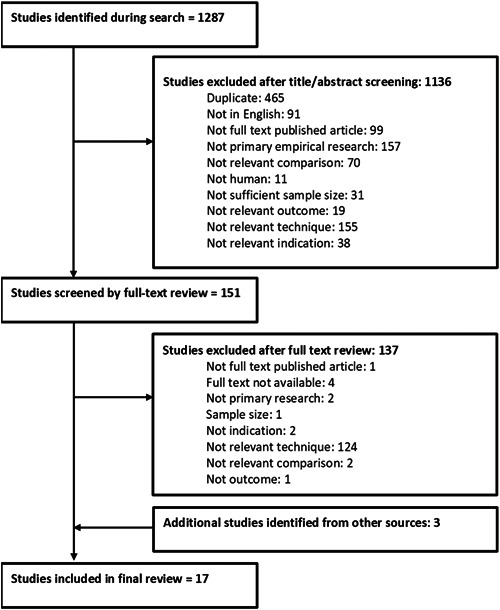
PRISMA diagram for the review.

The search strategy identified 1287 articles for consideration. After applying the inclusion and exclusion criteria, 17 articles were left as being relevant to the research question (Figure 1). Five articles identified in the databases searches^24^, ^25^, ^26^, ^27^, ^28^ may appear to meet inclusion criteria; however, these studies were not deemed to have implemented or solely implemented CIT.

### Study Characteristics

Key characteristics for each included study are presented in Table 1. The included studies consisted of 3 RCTs using a with‐in patient design,^29^, ^30^, ^38^ 5 RCTs using a between‐patient design,^32^, ^33^, ^34^, ^41^, ^45^ 1 prospective cohort study,^40^ and 8 retrospective cohort studies.^31^, ^35^, ^36^, ^37^, ^39^, ^42^, ^43^, ^44^ Across the 17 studies, a total of 1996 patients underwent CIT. The comparator arms included total tonsillectomy using plasma ablation (9 studies,^29^, ^30^, ^31^, ^34^, ^35^, ^36^, ^40^, ^41^, ^43^ n = 1100), electrocautery (4 studies,^32^, ^33^, ^38^, ^45^ n = 155), cold steel (2 studies,^37^, ^44^ n = 77), electrocautery or plasma ablation (1 study,^42^ n = 1800), any nonplasma ablation device (1 study,^35^ n = 1216) and nondefined device (1 study,^39^ n = 217).

**Table 1:** Study, Surgery, and Patient Characteristics of Included Studies

	Study design	Control procedure	Indication	Sample size	Age, y^a^
Arya et al^29^	RCT^b^	Plasma ablation	Infective	14	31.9 (12.1)
Arya et al^30^	RCT^b^	Plasma ablation	Infective	18	9 (range 3‐14)
Braverman et al^31^	Retrospective cohort	Plasma ablation	Obstructive with (control) or without (experimental) history of recurrent tonsillitis	Experimental 43 Control 37	Median 4 (range 2‐8) Median 5 (range 2‐17)
Chan et al^32^	RCT^c^	Electrocautery	Obstructive with no history of recurrent tonsillitis	Experimental 27 Control 28	6.4 (2.8) 5.9 (2.2)
Chang et al^33^	RCT^c^	Electrocautery	Obstructive with no history of recurrent tonsillitis	Experimental 52 Control 49	6.4 (3.4) 6.2 (3.4)
Chang et al^34^	RCT^c^	Plasma ablation	Obstructive with no history of recurrent tonsillitis	Experimental 34 Control 35	6.2 (3.3) 6.1 (2.9)
Divi et al^35^	Retrospective cohort	A. Plasma ablation B. Nonplasma ablation	Not stated	Experimental 303 Control A 239 Control B 1216	Not stated
Duarte et al^36^	Retrospective cohort	Plasma ablation	Obstructive and/or infective	Experimental 157 Control 258	6.7 (range 2‐18)
Friedman et al^37^	Retrospective cohort	Cold steel	Obstructive without history of recurrent tonsillitis	Experimental 50 (adult 15, pediatric 35) Control 50 (adult 10, pediatric 40)	Adults 31.1 (13.1) Pediatric 6.3 (2.8) Adult 27.2 (9.2) Pediatric 4.2 (1.4)
Hall et al^38^	RCT^b^	Electrocautery	Obstructive and/or infective	28	Adult, not stated
Heward et al^39^	Retrospective cohort	Not defined	Obstructive	Experimental 281 Control 217	Pediatric, not stated
Junaid et al^40^	Prospective cohort	Plasma ablation	Obstructive and/or infective	Experimental 23 Control 78	Pediatric, not stated
Lu et al^41^	RCT^c^	Plasma ablation	Obstructive and/or infective	Experimental 48 Control 42	5.3 (1.5)
Mukerji et al^42^	Retrospective cohort	Electrocautery or plasma ablation	Obstructive and/or infective	Experimental 467 Control 1800	6.23 (3.43) 6.82 (3.54)
Naidoo et al^43^	Retrospective cohort	Plasma ablation	Obstructive and/or infective	Experimental 351 (adult 36, pediatric 315) Control 379 (adult 44, pediatric 335)	7.0 (0.8‐74.3) 6.9 (0.7‐66.8)
Tremlett et al^44^	Retrospective cohort	Cold steel	Obstructive and/or infective	Experimental 47	3.5 (95% CI 3.2‐3.7)
				Control 27	3.4 (95% CI 3‐3.7)
Wilson et al^45^	RCT^c^	Electrocautery	Obstructive with no history of recurrent tonsillitis	Experimental 53	Median 5.8
				Control 50	Median 6.1

Abbreviation: RCT, randomized controlled trial.

^a^ Unless stated values are mean (standard deviation).

^b^ Within subject control design.

^c^ Between subject control design.

Eleven studies included pediatric patients,^30^, ^31^, ^32^, ^33^, ^34^, ^36^, ^39^, ^40^, ^41^, ^42^, ^44^ 3 included both adults and pediatric populations,^37^, ^43^, ^45^ 2 studies included adult patients,^29^, ^38^ and 1 did not report on age.^35^ The primary indication for all tonsillectomy procedures was obstructive (without history of recurrent infective indications) in 6,^32^, ^33^, ^34^, ^37^, ^39^, ^45^ infective in 2,^29^, ^30^ and mixed obstructive and/or infective in 7 studies.^36^, ^38^, ^40^, ^41^, ^42^, ^43^, ^44^ One study determined surgical procedure based on indication, with patients receiving CIT for obstructive indications without a history of recurrent tonsillitis and total tonsillectomy with a history of recurrent tonsillitis.^31^ One study did not identify indication for surgery.^35^

### Risk of Bias

Risk of bias for included studies are presented in Tables 2 and 3. The largest threat to bias in RCTs was a lack of intention‐to‐treat analysis, although is unlikely to be relevant based on individual study methods, and limited detail on randomization and concealment. The largest threat to bias evident across the observational studies was a lack of blinding of outcome assessors to treatment allocation and potential for confounding characteristics between treatment groups.

**Table 2:** Risk of Bias for Included Randomised Controlled Trials

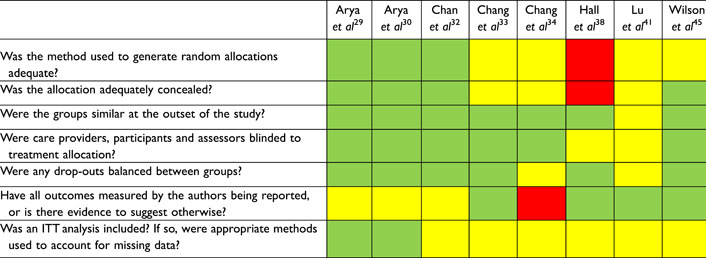

Green = yes; Yellow = partially or unclear; Red = no.

**Table 3:** Risk of Bias for Included Cohort Studies

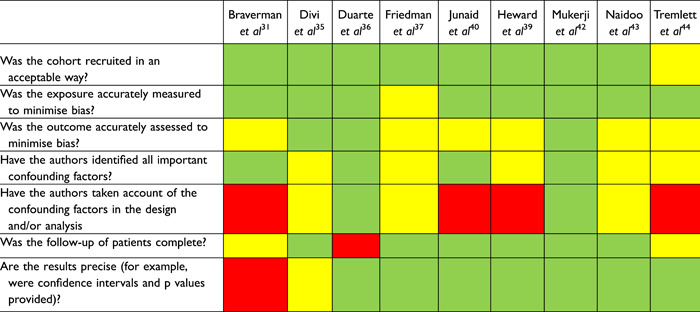

Green = yes; Yellow = partially or unclear; Red = no.

### Postoperative Pain and Analgesia Use

**Figure 2:**
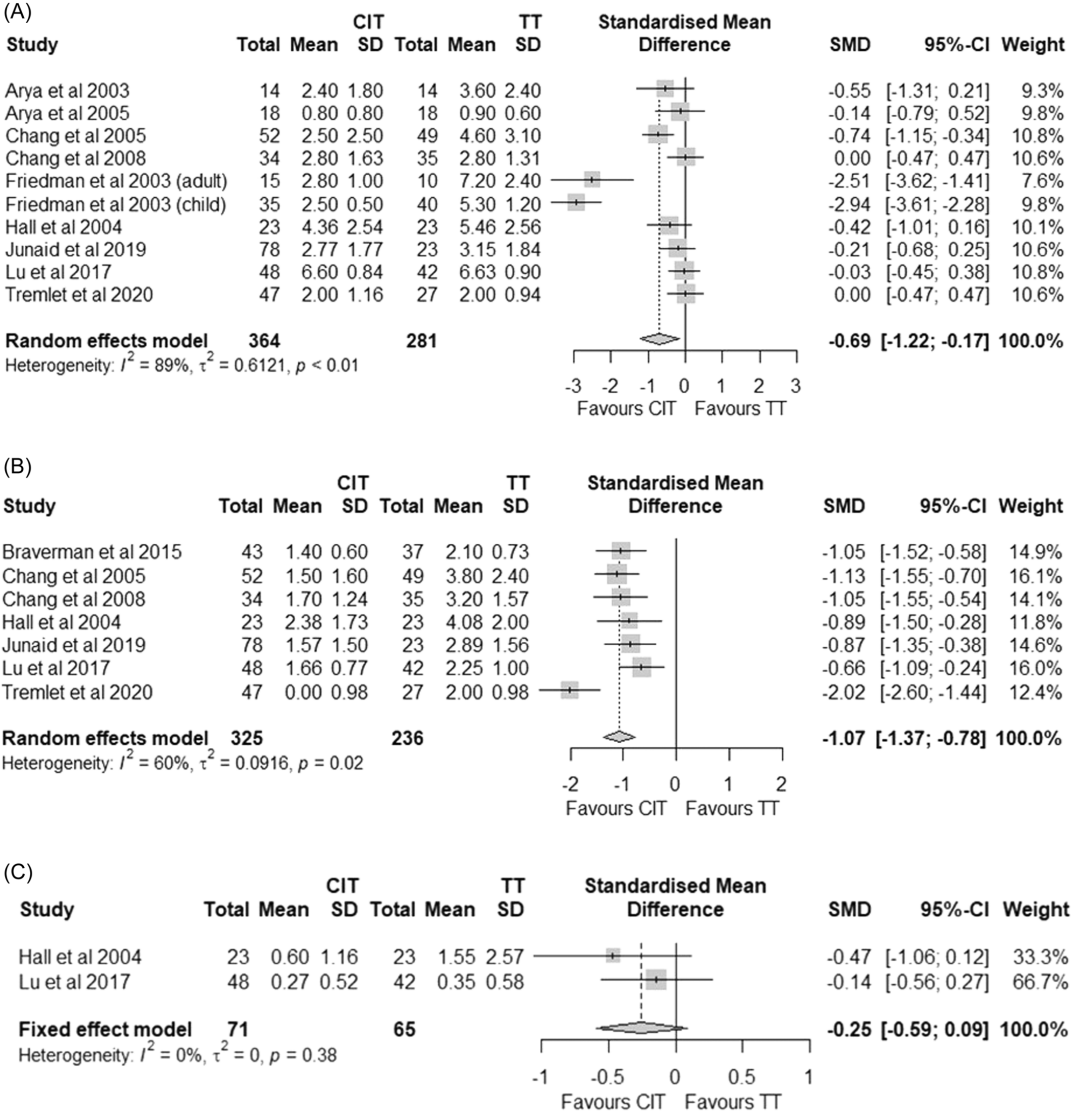
Absolute pain at 1 day (A), 1 week (B), and 2 weeks (C). CIT, intracapsular tonsillectomy using plasma ablation; TT, total tonsillectomy.

Eleven studies reported on absolute pain scores, and all observed statistically significantly lower pain score at earlier time‐points with CIT.^29^, ^30^, ^31^, ^33^, ^34^, ^36^, ^37^, ^38^, ^40^, ^41^, ^44^, ^45^ Nine studies reported on absolute pain over the first 24 hours,^29^, ^30^, ^33^, ^34^, ^37^, ^38^, ^40^, ^41^, ^44^ and meta‐analysis identified pain scores to be statistically significantly lower following CIT (Figure 2A; SMD −0.69; 95% CI −1.22 to −0.17; *p* = .0092). Seven studies reported on absolute pain at 1 week (5‐8 days)^31^, ^33^, ^34^, ^38^, ^40^, ^41^, ^44^ and meta‐analysis identified these pain scores to be statistically significantly lower following CIT (Figure 2B; SMD −1.07; 95% CI −1.36 to −0.78; *p* < .0001). Two studies reported on pain at 2 weeks^38^, ^41^ and meta‐analysis identified these pain scores to not be statistically significantly different with CIT (Figure 2C; SMD −0.25; 95% CI −0.59 to 0.09; *p* = .14).

**Figure 3:**
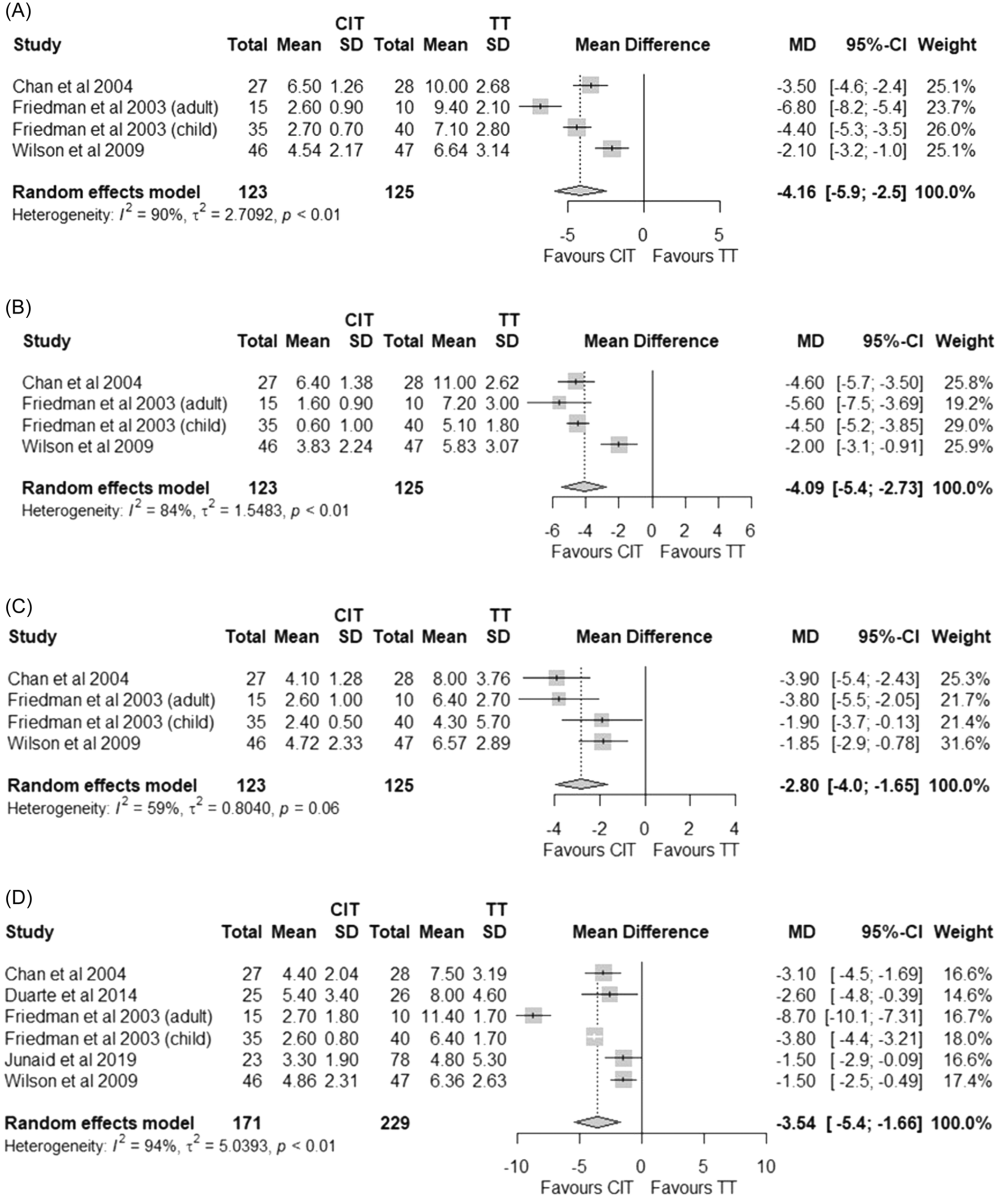
Time (days) taken to pain free (A), cessation of analgesia use (B), return to normal activity (C), and diet (D). CIT, intracapsular tonsillectomy using plasma ablation; TT, total tonsillectomy.

Three studies reported on time to pain free, all observing statistically significantly faster time to pain free following CIT compared to total tonsillectomy.^32^, ^37^, ^45^ Meta‐analysis identified the time to pain free across these studies to be statistically significantly faster with CIT, with a mean difference of 4.2 days (Figure 3A; 95% CI −5.9 to −2.5 days; *p* < .0001). These same studies reported on the time on analgesia and meta‐analysis identified statistically significantly less time on analgesia following CIT by a mean of 4.1 days (Figure 3B; 95% CI −5.4 to −2.7 days; *p* < .0001).

In addition to the studies and data amenable to meta‐analysis, Braverman et al^31^ reported that patients were less likely to need analgesia following CIT than total tonsillectomy (49% vs 73%; *p* < .05) and if a patient did require analgesia they were also less likely to need multiple doses with CIT. This was also observed by Mukerji et al (0.86% vs 14%; *p* < .001).^42^ Chang et al^33^ identified that at 5 to 6 days postoperatively patients who underwent CIT were on statistically significantly lower doses of paracetamol than following total tonsillectomy using electrocautery (*p* < .0005); however, Chang et al^34^ reported no statistically significant difference in analgesia use between CIT and total tonsillectomy using plasma ablation. Tremlett et al^44^ also observed no statistical differences in the doses of morphine following CIT and total tonsillectomy using cold steel.

### Return to Normal

Three studies reported on time taken to return to normal activity and all observed statistically significantly faster times following CIT than total tonsillectomy.^32^, ^37^, ^45^ Meta‐analysis identified this to occur 2.8 days faster with CIT than total tonsillectomy (Figure 3C; 95% CI −4.0 to −1.6 d; *p* < .0001). Five studies reported on time taken to return to normal diet^32^, ^36^, ^37^, ^40^, ^45^ and meta‐analysis identified a statistically significant faster return to normal diet with CIT than total tonsillectomy (Figure 3D, MD −3.5 days; 95% CI −5.4 to −1.7 days; *p* = .0002).

In addition to studies amenable to meta‐analysis, 3 studies reported on the proportion of patients returning to normal activity,^33^, ^34^ and 4 returning to normal diet,^31^, ^33^, ^34^, ^44^ at a given time and all except 1 study^44^ observed a higher proportion of patients to have returned following CIT than total tonsillectomy. Tremlett et al^44^ reported a greater percentage to return to normal diet and activity with CIT at 1, 3, and 7 days postoperatively; however, statistical significance was only reached for return to normal diet at 1 day postoperatively.

### Efficacy

Three studies reported on the efficacy of CIT in comparison to total tonsillectomy. Braverman et al^31^ reported similar mean Obstructive Sleep Apnea questionnaire scores following CIT and total tonsillectomy using plasma ablation (25.5 and 24.6, respectively). Furthermore, all children were free from obstructive sleep apnea symptoms; however, 4.7% (2 out of 43) and 13.5% (5 out of 37) of children were still snoring following the CIT and total tonsillectomy, respectively. Mukerji et al identified similar postoperative improvements in the apnea hyponea index following CIT and total tonsillectomy in a subgroup of patients who underwent sleep study.^42^ Chan et al^32^ reported no statistical differences in the improvements in obstructive symptoms between CIT and total tonsillectomy using electrocautery at 3‐ or 12‐months postsurgery.

### Complications

**Figure 4:**
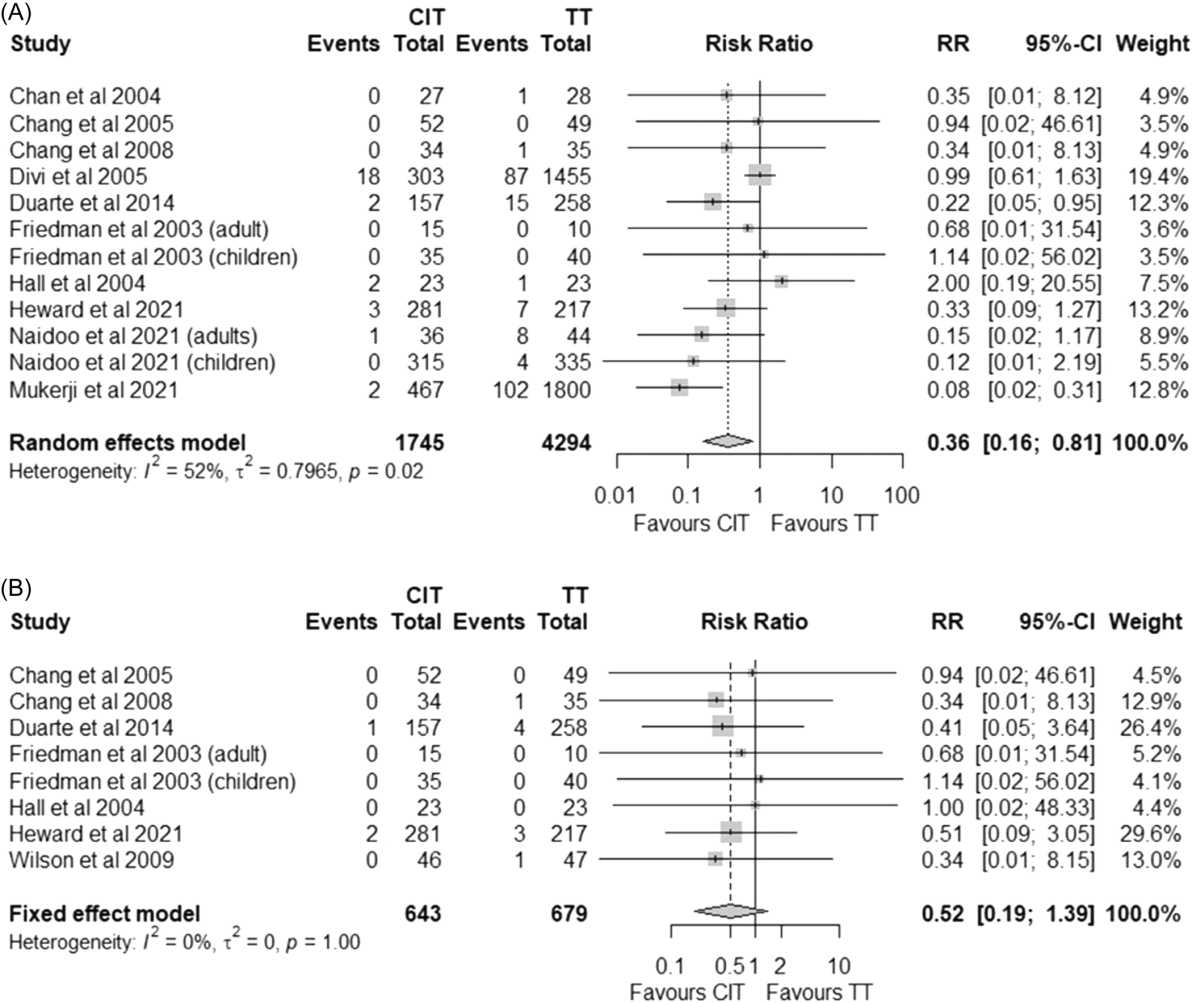
All posttonsillectomy hemorrhage events (A) and those requiring return to surgery (B). CIT, intracapsular tonsillectomy using plasma ablation; TT, total tonsillectomy.

Thirteen studies reported on PTH^29^, ^30^, ^32^, ^33^, ^34^, ^35^, ^36^, ^37^, ^38^, ^39^, ^42^, ^44^, ^45^; however, two of these studies did not provide sufficient information to determine which procedure the observed event was associated to^29^, ^30^ and 1 study could only attribute PTH requiring surgical management to a specific procedure and not all PTH events.^45^ Meta‐analysis revealed a statistically significant lower risk of PTH events with CIT (Figure 4A; RR 0.36; 95% CI 0.16 to 0.81; *p* = .0131); however, no statistical differences were identified when considering PTH requiring surgical management (Figure 4B; RR 0.52; 95% CI 0.19 to 1.39; *p* = .19).

Eleven studies reported on complications other than PTH (Supplemental Table S1, available online)^31^, ^32^, ^33^, ^34^, ^36^, ^37^, ^40^, ^42^, ^43^, ^44^, ^45^; however, of these Duarte et al^36^ did not provide sufficient information to associate these to a specific procedure. There were no consistent differences across the studies between the procedures for total complication rates or specifically dehydration, nausea, vomiting, and fever rates. Junaid et al^40^ reported a higher rate of patients seeing their general practitioner in the 8 days following total tonsillectomy using plasma ablation compared to CIT (24% vs 15%) and that the majority of these visits resulted in a course of antibiotics. Mukerji et al^42^ also observed a lower return to the emergency centre with CIT (3% vs 11%) but Tremlett et al^44^ identified no differences in seeking medical discharge following surgery (19% vs 22%). Mukerji et al^42^ identified 5 patients (1.1%) who received CIT to undergo a revision procedure and Naidoo et al^43^ reported 1 adult patient (0.3% of all patients, 2.7% of adult patients) to undergo reoperation following a peritonsillar abscess 6 months postsurgery. Lu et al^41^ reported no regrowth of the tonsil or complaints of tonsilitis symptoms at least 1 year following surgery. Chan et al^32^ reported 2 and 1 cases (out of the 27 patients undergoing CIT) to have >10% of tonsil tissue remaining, suggestive of re‐growth, at 3 and 12 months following surgery, respectively. However, these two patients at 3 months reported improvement in 11 and 12 obstructive symptoms (out of 13) and the patient at 12 months only reported more frequent restless sleep but no other symptoms compared to before surgery.

### Sensitivity Analyses

Sensitivity analyses were performed to determine the robustness of the conclusions reached from each analysis presented. Consideration of the alternative meta‐analysis model, leave‐one‐out analysis, and substitution of equivalent data provided by parent/guardian/caregiver over patient did not alter the observed statistical significance or overall conclusions for any of the outcomes assessed. Consideration of only pediatric data did not alter the observed statistical significance or overall conclusions for any of the outcomes assessed except for pain at 1 day postoperative (SMD −0.56; 95% CI −1.19 to 0.07; *p* = .08).

## Discussion

The main observation of this systematic literature review was that CIT resulted in reduced patient morbidity in comparison to total tonsillectomy in terms of: lower absolute pain; faster time to pain free, normal diet and normal activity; and less time on analgesia. CIT also resulted in a lower risk of PTH; however, only total rates and not those requiring return to surgery reached statistical significance.

CIT was found to allow patients to return to their normal activities on average 3 to 4 days (~40%‐50%) faster than following total tonsillectomy. In addition, absolute pain was reported to be significantly lower both a day and a week following CIT compared to total tonsillectomy, with the greatest difference occurring a week postoperatively. No significant differences were identified between CIT and total tonsillectomy two weeks postoperatively; this is perhaps not unexpected as most patients across all studies had become pain free prior to 2 weeks. Total tonsillectomy results in the removal of the tonsil capsule and consequently the exposure of the underlying muscle tissue, blood vessels, and nerves within the tonsillar fossae leading to the severe and prolonged pain and morbidity observed. CIT leaves the tonsil capsule in place, which has been proposed to act as a “biologic dressing” protecting the pharyngeal muscles, blood vessels and nerves, minimizing the postoperative pain and morbidity.^41^

One of the main complications associated with total tonsillectomy is PTH, with a systematic review reporting a mean total PTH rate of 3.4%, readmission rate associated with PTH of 1.8% and reoperation associated with PTH of 1.6%.^46^ CIT was identified to result in a statistically significant 64% reduction in PTH events compared to total tonsillectomy (*p* = .0131); however, PTH events requiring surgical management between CIT were not statistically different between techniques (RR 0.52; *p* = .19). This is likely to be an artifact of the relatively low sample size inherent to the comparative studies included in this systematic literature review combined with relatively low events resulting in a type II statistical error. Recent large case‐series of patients undergoing CIT have reported very low PTH rates of 0.5%^9^ and PTH requiring surgical management rates of 0.2%^9^ and 0%.^8^ These case‐series demonstrate rates which are substantially lower than those previously reported following total tonsillectomy.^46^ Across all studies, there was minimal detail on the severity of the bleed and the definition and reporting of PTH showed substantial variability including criteria of any case of PTH, cases requiring readmission, cases requiring medical intervention, and cases requiring surgical intervention.

The effectiveness of CIT in resolving the original indication for surgery was shown to be similar to total tonsillectomy in the 3 studies reporting such an outcome in this systematic literature review.^31^, ^32^, ^42^ Efficacy in these studies included only patients undergoing surgery for obstructive indications; however, a large noncomparative case‐series of patients including those with obstructive, infective or obstructive and infective indications who underwent CIT (n = 1257) observed significant improvements in T‐14 scores.^9^ Similarly, a second case‐series of 80 patients undergoing CIT for infective indications observed significant improvements in T‐14 scores.^10^

Perhaps the largest objection to uptake of CIT, and intracapsular procedures in general, is the risk of needing a second surgery, due to regrowth of the tonsils and return of obstructive symptoms or remnant tonsil tissue being susceptible to further infection. In the present systematic literature review, the majority of studies did not include a sufficient follow‐up period to capture regrowth and revision surgery; however, Lu et al^41^ identified no cases of tonsil regrowth or infection in the year following CIT while Chan et al^32^ reported 1 patient (5%) who presented regrowth, but remained asymptomatic and did not require reoperation, after 1 year and Mukerji et al^42^ identified 5 patients (1.1%) who underwent revision tonsillectomy. In support of a low risk of a second tonsil procedure following CIT, Amin et al^9^ reported a revision rate of 2.6% over a median 3.9 years follow‐up in a case‐series of 1257 patients and Varadharajan et al^10^ reported zero revisions with a mean follow‐up of 1.1 years in a case series of 80 patients who underwent CIT for infective indications. When selecting a surgical technique, it is important to consider this risk of regrowth and need for an elective second procedure against the risk of PTH, which can be a potentially life‐threatening complication requiring emergency readmission and surgical intervention.^47^

The included studies in this systematic literature review tended to be of a smaller comparative design but no study was identified to be at high risk of bias. The most common threat to bias in RCTs was a lack of clarity on the method of randomization and concealment combined with lack of an intention‐to‐treat analysis. An intention‐to‐treat analysis is unlikely to contribute towards bias in these studies due to high patient follow‐up combined with no known deviation from the surgical procedure. The largest threat to bias in observational studies were lack of blinding to outcome assessors, particularly for subjective outcomes such as pain and return to normal, due to the assessor (typically the parent/guardian/caregiver) being informed of the benefit and risk of the surgical procedure. Meta‐analysis for these subjective outcomes included studies from both RCTs and observational studies; observation of individual studies on forest plots showed similar outcomes across the two study designs. A second common threat to bias was the allocation of surgical techniques based on indications, with CIT typically performed on patients with obstructive indications only.^48^ These between‐group differences were often not acknowledged by the authors or considered within analysis; however, research suggests that these reported are unlikely to be affected by indication.^9^ Sensitivity analysis identified that the statistical observations remained when leaving 1 study out of analysis and when considering only pediatric data, suggesting observations are robust and likely relevant to the broad age population across included studies.

The higher tendency to use CIT on patients with obstructive indications could be related to a view that the efficacy of resolving infective symptoms, and the potential resulting need for revision, is not favorable due to leaving remnant tonsil tissue.^11^ Recent literature has shown high efficacy and low revision rates when using CIT in infective indications.^9^, ^10^ The follow‐up in many studies was relatively short (<1 month), which is sufficient to establish the resolution of symptoms, return to normal state and majority of postoperative complications; however, this may not fully capture the return of symptoms. Therefore, future research should focus on greater consideration of patients with infective indications undergoing CIT combined with longer follow‐up to capture fully the efficacy of the procedures. The greater proportion of studies investigating tonsillectomy procedures for obstructive indications is also likely reflective of current practice where obstructive indications are the most common reason for tonsillectomy.^49^

The clinical implications of these findings are multifaceted. The observed rise in tonsillar surgery for obstructive indications has meant that children undergoing this type of surgery are younger than has traditionally been the case. This has led to concerns with regard to the risks associated with bleeding in smaller children. The existence of an effective surgical technique with a decreased risk profile is a highly promising prospect when considering interventions in younger children. Decreased pain, compared with traditional, total tonsillectomy techniques is likely to allow earlier discharge from hospital resulting in more efficient delivery of services and has the potential to reduce reliance on opiate analgesia in the initial post‐operative period. The avoidance or minimization of the use of drugs with known respiratory suppressant effects is likely to have significant benefits when managing children with sleep‐disordered breathing. Reduction in pain in the days following surgery and a faster return to normal function has significant implications for patients and parents. The ability to return to daycare or school after surgery means that parents and caregivers are able to return to work more quickly with benefits not only to them but to society at large.

## Conclusions

This systematic literature review demonstrates that CIT has similar efficacy in managing indications for tonsil surgery to total tonsillectomy while significantly reducing the post‐operative morbidity experienced by patients, allowing them to return to their normal life faster. In addition, the evidence suggests that CIT did not result in additional complications compared to total tonsillectomy and, in most cases, CIT tended to provide a lower rate of complications including a significantly reduced risk of PTH. These findings would be further strengthened with additional large RCTs with increased inclusion of patients with infective indications and longer follow‐up to fully establish the short‐ and long‐term safety and effectiveness of the procedures.

## Author Contributions

**Matthew J. Sedgwick**, conception/design of work; data acquisition, analysis and data interpretation; drafting the work; read and approved final manuscript; **Christopher Saunders**, Conception/design of work; data interpretation; critical review of manuscript; read and approved final manuscript; **Neil Bateman**, Data interpretation; critical review of manuscript; read and approved final manuscript.

## Disclosures

### Competing interests

Neil Bateman receives consulting fees from Smith+Nephew. Matthew J. Sedgwick and Christopher Saunders are both employees of Smith+Nephew.

### Sponsorships

None.

### Funding source

None.

## Supporting information

Supplementary information.
