# Supplementary material for: Intracapsular Tonsillectomy Using Plasma Ablation Versus Total Tonsillectomy: A Systematic Literature Review and Meta‐Analysis
Source: OTO Open. 2023 Feb 17;7(1):e22. doi: 10.1002/oto2.22 (PMC10046729; doi:10.1002/oto2.22)
Supplement: Supplementary file 1 — Supplementary information. [file OTO2-7-e22-s001.docx]

Supplemental Table 1. Complications other than post-tonsillectomy haemorrhage

|  | **Procedure** | **N** | **Complications** |
| --- | --- | --- | --- |
| Braverman *et al* ^38^ | CIT | 43 | 0 intraoperative or post-operative complications |
|  | Total – Plasma ablation | 37 | 5 intraoperative or post-operative complications (most of them dehydration)* |
| Chan *et al* ^32^ | CIT | 27 | 4 patients experienced nausea  0 patients experienced dehydration (requiring re-admission)  4 patients presented eschar  2 patients with residual tonsillar tissue >10% at 3 month (1 improved in 12/13 obstructive symptoms and 1 improved in 11/13 symptoms)  1 patient with residual tonsillar tissue >10% at 12 month (reporting worsening in 1/13 obstructive symptoms)  10 patients reported worsening in at least 1/13 obstructive symptoms at 3 month |
|  | Total - Electrocautery | 28 | 14 patients experienced nausea*  1 patients experienced dehydration (required re-admission)  13 patients presented eschar*  0 patients with residual tonsillar tissue >10% at 3 month  0 patient with residual tonsillar tissue >10% at 12 month (reporting worsening in 1/13 obstructive symptoms)  6 patients reported worsening in at least 1/13 obstructive symptoms at 3 month |
| Chang *et al* ^33^ | CIT | 52 | 6 nausea  0 dehydration (requiring re-admission)  11 vomiting |
|  | Total - Electrocautery | 49 | 7 nausea  1 dehydration (requiring re-admission)  8 vomiting |
| Chang *et al* ^34^ | CIT | 34 | 0 dehydration (requiring re-admission)  7 nausea  6 vomiting |
|  | Total – Plasma ablation | 35 | 0 dehydration (requiring re-admission)  1 temporary velopharyngeal insufficiency symptoms that resolved after 2 months  8 nausea  8 vomiting |
| Duarte *et al* ^40^ | CIT | 258 | *Insufficient information was provided to associate these complications to a procedure*.  1 patient with intraoperative complication which involved reintubation after extubation and did not result in any consequences post-operatively.  2 patients with early postoperative complications (1 hematemesis and 1 post-extubation laryngospasm and post-obstructive pulmonary edema), both recovered unremarkably and neither required a return to the operating room.  3 patients with late non-hemorrhagic postoperative complications (1 nausea and vomiting, 1 fever along with nausea and vomiting, and 1 epistaxis unrelated to tonsillectomy). |
|  | Total – Plasma ablation | 157 |  |
| Friedman *et al* ^41^ | CIT | 50 | 0 intraoperative or postoperative complications  0 dehydration  0 hospitalization or intravenous fluids for >23 h  0 re-admissions  Post-operative fevers did occur but required no specific treatment |
|  | Total – Cold steel | 50 | 0 intraoperative or postoperative complications  0 dehydration  0 hospitalization or intravenous fluids for >23 h  0 re-admissions  Post-operative fevers did occur but required no specific treatment |
| Junaid *et al* ^37^ | CIT | 23 | 3 reviewed by GP  2 required antibiotics |
|  | Total – Plasma ablation | 78 | 19 reviewed by GP  14 required antibiotics |
| Lu *et al* ^35^ | CIT | 48 | 0 regrowth of the tonsils was found by physical examination  0 patients complained of tonsilitis symptoms post surgery |
|  | Total – Plasma ablation | 42 | 0 regrowth of the tonsils was found by physical examination  0 patients complained of tonsilitis symptoms post surgery |
| Mukerji *et al* ^44^ | CIT | 467 | 12 emergency centre return  5 revision tonsillectomy |
|  | Total – Plasma ablation or Electrocautery | 1800 | 191 emergency centre return  0 revision tonsillectomy |
| Naidoo *et al* ^43^ | CIT | 351 | 1 adult patient revision surgery following peritonsillar abscess 6 months post surgery |
|  | Total – Plasma ablation | 379 | - |
| Tremlett *et al* ^42^ | CIT | 47 | 9 failing to meet discharge criteria (1 inadequate oral intake, 8 poor overnight oxygen saturation)  9 seeking medical advice following discharge (mainly from GP) |
|  | Total – Cold Steel | 27 | 6 failing to meet discharge criteria (all due to inadequate oral intake)  6 seeking medical advice following discharge (mainly from GP) |
| Wilson *et al* ^36^ | CIT | 46 | 23 patients had a complication (including 12 post-op fever, 9 voice change, 3 ear pain, 5 neck pain, 0 emergency room visit, 0 rebleeding requiring surgery and 1 intraoperative placement of sutures to control hemostasis).  17 patients had one complication and 6 had ≥2 complications |
|  | Total - Electrocautery | 47 | 34 patients had a complication (including 13 post-op fever, 12 voice change, 8 ear pain, 4 neck pain, 4 emergency room visit, 1 rebleeding requiring surgery and 7 intraoperative placement of sutures to control hemostasis).  20 patients had one complication and 14 had ≥2 complications |

* = Within study statistically significant different between procedures
